# Long-term prognosis of vascular access in hemodialysis patients with systemic lupus erythematosus: a retrospective cohort study

**DOI:** 10.1038/s41598-021-92005-5

**Published:** 2021-06-15

**Authors:** Fan-Yu Chen, Chun-Fan Chen, Ann Charis Tan, Chia-Hao Chan, Fu-An Chen, Wen-Sheng Liu, Tz-Heng Chen, Shuo-Ming Ou, Szu-Yuan Li, Ming-Tsun Tsai, Yung-Tai Chen, Chih-Ching Lin

**Affiliations:** 1 School of Medicine, National Yang Ming Chiao Tung University, Hsinchu, Taiwan; 2 School of Medicine, National Yang-Ming University, Taipei, Taiwan; 3 Division of Nephrology, Department of Medicine, Taipei Veterans General Hospital, No. 201, Section 2, Shih-Pai Road, Beitou District, Taipei, 11217 Taiwan; 4 Division of Nephrology, Department of Internal Medicine, National Yang Ming Chiao Tung University Hospital, Yilan, Taiwan; 5 Division of Nephrology, Department of Medicine, Taipei City Hospital Zhongxing Branch, Taipei, Taiwan; 6 Institute of Food Safety and Health Risk Assessment, National Yang Ming Chiao Tung University, Hsinchu, Taiwan; 7 Institute of Food Safety and Health Risk Assessment, National Yang-Ming University, Taipei, Taiwan; 8 College of Science and Engineering, Fu Jen Catholic University, New Taipei City, Taiwan; 9 University of Taipei, Taipei, Taiwan; 10 Division of Nephrology, Department of Medicine, Fenglin Branch, Taipei Veterans General Hospital, Hualien, Taiwan; 11 Division of Nephrology, Department of Internal Medicine, Fuyou Branch, Taipei City Hospital Heping, Taipei, Taiwan

**Keywords:** Haemodialysis, End-stage renal disease, Systemic lupus erythematosus

## Abstract

Patients with systemic lupus erythematosus (SLE) have a higher risk of vascular complications. This retrospective cohort study aimed to analyze the differences in the risk of arteriovenous fistula or graft (AVF/AVG) dysfunction in hemodialysis patients with and without SLE from Taiwan’s National Health Insurance Database over a 10-year period. AVF/AVG dysfunction is defined as the occurrence of the first episode of intervention after vascular access creation. A total of 1366 HD patients with SLE had higher incidence rates of AVF/AVG dysfunction than 4098 non-SLE HD patients in the following 4 periods: (1) after 1 year (incidence rates = 15.21% and 13.01%, respectively; subdistribution hazard ratio (SHR) = 1.16; P = 0.007), (2) 1st-to-10th-year period (15.36% and 13.25%; SHR = 1.16; P = 0.007), (3) 5th-to-10th-year period (11.91% and 8.1%; SHR = 1.42; P = 0.003), and (4) overall period (23.53% and 21.66%; SHR = 1.09; P = 0.027). In conclusion, there were significantly higher incidence rates of AVF/AVG dysfunction in SLE patients during the long-term follow-up period. Vascular access function should be monitored regularly by clinical examinations, especially after 1 year and during 5 to 10 years, to improve AVF/AVG patency and dialysis adequacy in SLE patients undergoing maintenance hemodialysis.

## Introduction

Systemic lupus erythematosus (SLE) is a systemic autoimmune disease that has a worldwide prevalence ranging from 0.3 to 23 per 100,000 person-years, affecting many of different age, racial, and ethnic groups^1^. Asian SLE patients manifest higher rates of renal involvement (50–60%) compared to Caucasian patients (30–38%) and are often associated with a greater risk of severe renal disease^2^. There were approximately 612.8 SLE cases per 100,000 patient-years that progressed to end-stage renal disease (ESRD) and received hemodialysis (HD) based on the National Health Insurance Research Database (NHIRD) in Taiwan between 2000 and 2008^3^.

Vascular diseases are commonly observed in SLE patients. Vascular access dysfunction, involving either arteriovenous fistulas (AVF) or arteriovenous grafts (AVG), is an important factor that not only determines the quality of HD, but also has a crucial impact on morbidity and mortality. Prolonging access patency and limiting the complications of a functioning access require interprofessional collaborative practice.

Previous research pointed out that SLE patients on HD are more probable to develop vascular access thrombosis^4^. Antiphospholipid syndrome (APS), which is the association between thrombosis and/or pregnancy morbidity with the presence of antiphospholipid (aPL) antibodies, may have an impact on SLE presentation, management, and prognosis. There are 30–40% of SLE patients who have tested positive for aPL. Compared to SLE patients without aPL, those with aPL have a higher prevalence of vascular thrombosis and pregnancy morbidity, poorer quality of life, and higher risk of organ damage^5^. Theoretically, SLE patients may have an increased risk of vascular access patency loss even from the time of AVF/AVG creation. However, it is still uncertain whether or not the SLE disease itself has an impact on vascular access patency. There are studies conducted that were focused only on the short-term outcome (within 1 year) of vascular access patency in SLE-ESRD patients, and the conclusion is still very controversial^4,6^. So far, little is known whether or not there is a difference in the rate of AVF/AVG dysfunction between SLE patients and non-SLE patients during the long-term follow-up period (after 1 year and onwards). Therefore, the study aims to investigate the long-term dysfunction rate of AVF/AVG in HD patients with and without SLE.

## Methods

### Study design and patient selection

In this retrospective cohort study, data were obtained from Taiwan’s NHIRD. Since 1995, all citizens and residents in Taiwan are provided with compulsory universal health insurance. The program provides full coverage for renal replacement therapy for patients with ESRD. Healthcare institutions are then required to submit computerized claim documents for renal replacement therapy to the National Health Insurance Administration. Taiwan’s NHIRD is a population-based data source for producing real-world data to help make diagnostic decisions and health care policies, which covers almost all of the inpatient and outpatient medical records for Taiwan’s 23 million residents. Information such as patient identification number, birthday, gender, dates of hospital admission and discharge, healthcare institutions providing services, ICD-9-CM/ICD-10-CM diagnostic and procedure codes, and outcomes, among many other data, are stored in this database.

The study was carried out in accordance with the Helsinki Declaration (edition 6, revised 2000) and was approved by the Institutional Review Board of Taipei Veterans General Hospital (2020-09-018BC). The need of informed consent was waived by the review board since the dataset was encrypted and de-identified.

Data from the NHIRD were collected for HD patients in Taiwan between 2000 and 2011. The patients were divided into two groups (SLE and non-SLE group). The exclusion criteria of the study are as follows: (1) under 20 years old, (2) undergoing peritoneal dialysis, (3) pregnant, (4) kidney transplant recipients, (5) had never initiated HD via AVF/AVG, (6) had never received a temporary or permanent double-lumen catheter placed before AVF/AVG creation, and (6) ineligible for the National Health Insurance catastrophic illness card (given to HD patients who require life-long renal replacement therapy).

The demographic data of the patients included in the analysis consisted of their age, gender, Charlson Comorbidity Index score, vascular access type, time from vascular access creation to HD initiation, medications (antiplatelet agents, angiotensin-converting enzyme inhibitor/angiotensin II receptor blocker, beta blocker, calcium channel blocker, statin, warfarin, steroids, hydroxychloroquine/chloroquine, and immunosuppressants), and comorbidities (diabetes mellitus, hypertension, myocardial infarction, heart failure, peripheral vascular disease, dementia, chronic pulmonary disease, dyslipidemia, cerebrovascular disease, valvular heart disease, and cancer).

AVF/AVG dysfunction is defined as the occurrence of the first episode of intervention (angioplasty, thrombectomy, or new AVF/AVG creation, etc.) after vascular access creation. The primary outcome in this study is the cumulative incidence rate of AVF/AVG dysfunction, which measures the occurrence of an intervention from the time of vascular access creation to the first episode of dysfunction within 3 months, 6 months, 1 year, 5 years, and 10 years. The secondary outcomes include the occurrence of major adverse cardiovascular events (MACE) (defined as the first occurrence of death from cardiovascular causes, non-fatal myocardial infarction, or non-fatal stroke), myocardial infarction, and ischemic stroke. SLE and non-SLE patients were also further stratified according to the presence of diabetes mellitus and hypertension and underwent additional subgroup analysis.

### Statistical analysis

Data analysis was performed using SAS software version 8.0 (SAS Institute, Cary, North Carolina, USA). Continuous variables were presented in mean ± standard deviation and examined using t-test. Categorical variables were presented in number and percentage and examined using chi-square test. All data were normally distributed. Using the same propensity score matching method in our previous study^7^, the propensity scores of the probability of SLE diagnosis were established using multivariate logistic regression, conditional on the baseline covariates (Supplementary Table 1). Three non-SLE patients were matched with a SLE patient that has a similar propensity score based on the nearest neighbor matching without replacement using calipers of width equal to 0.1 of the standard deviation of the logit of the propensity score. Survival curves indicating the cumulative incidence rate of AVF/AVG dysfunction were examined using the Cox regression model, Kaplan–Meier method, and log-rank test. The subdistribution hazard ratio was obtained from the Fine and Gray model. A value of P < 0.05 was considered significant.

## Results

**Figure 1:**
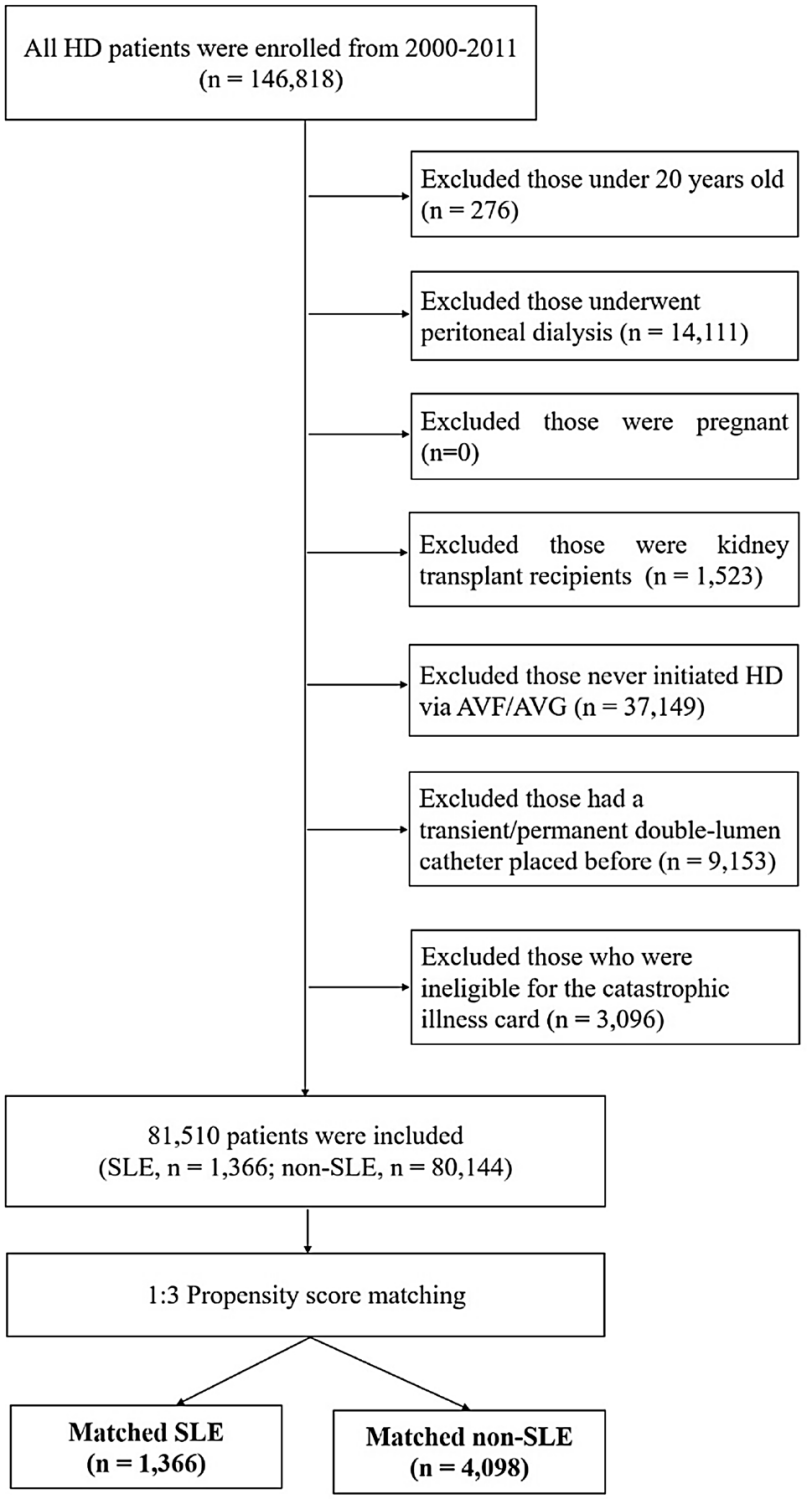
Flowchart of patient enrollment and propensity score matching.

A total of 146,818 HD patients were enrolled. However, 65,308 patients were excluded from the study for the following reasons: 276 were under 20 years old, 14,111 underwent peritoneal dialysis, 0 was pregnant, 1523 were kidney transplant recipients, 37,149 had never initiated HD via AVF/AVG, 9153 had a transient/permanent double-lumen catheter placed before AVF/AVG creation, and 3096 were not eligible for the catastrophic illness card. A total of 81,510 patients were selected, which comprised of 1366 SLE and 80,144 non-SLE patients. After implementing propensity score matching with a ratio of 1:3, 1366 SLE and 4098 non-SLE patients remained in the study. A flowchart in Fig. 1 summarizes the entire process.

The baseline characteristics of SLE and non-SLE patients shown in Table 1 were not found to be significantly different. The distribution of patients in the SLE and non-SLE group have similar values in terms of mean age (51 and 51.2 years old, respectively), gender percentage (23% males and 77% females; 25% males and 75% females, respectively), mean Charlson Comorbidity Index scores (6.0 for both groups), and the number of patients with AVF (1189 and 3577, respectively). The use of concomitant medications (except for warfarin, steroids, hydroxychloroquine/chloroquine, and immunosuppressants) and the presence of comorbidities were also similar in both groups.

**Table 1:** Baseline characteristics of patients.

Characteristics	SLE patients (N = 1366)	Non-SLE patients (N = 4098)	P
Age, years, mean (SD)	51.0 (16.8)	51.2 (15.2)	0.616
**Gender**			
Male	312 (22.84)	1011 (24.67)	0.171
Female	1054 (77.16)	3087 (75.33)	
CCI score, mean (SD)	6.0 (2.7)	6.0 (3.5)	0.894
AVF	1189 (87.04)	3577 (87.29)	0.815
**Concomitant medications**			
Antiplatelet agents^a^	445 (32.58)	1339 (32.67)	0.947
ACE inhibitor or ARB	514 (37.63)	1528 (37.29)	0.821
Beta blocker	570 (41.73)	1721 (42)	0.862
Calcium channel blocker	846 (61.93)	2526 (61.64)	0.847
Statin	179 (13.10)	484 (11.81)	0.205
Warfarin	108 (7.91)	101 (2.46)	< 0.001
Steroids	616 (45.1)	659 (16.08)	< 0.001
Hydroxychloroquine/chloroquine	522 (38.21)	57 (1.39)	< 0.001
Immunosuppressants	406 (3.37)	86 (2.1)	< 0.001
**Comorbidities**			
Diabetes mellitus	447 (32.72)	1336 (32.6)	0.933
Hypertension	1175 (86.02)	3521 (85.92)	0.928
Myocardial infarction	64 (4.69)	186 (4.54)	0.823
Heart failure	423 (30.97)	1241 (30.28)	0.635
Peripheral vascular disease	93 (6.81)	283 (6.91)	0.902
Dementia	33 (2.42)	102 (2.49)	0.880
Chronic pulmonary disease	597 (43.7)	1839 (44.88)	0.451
Dyslipidemia	579 (42.39)	1704 (41.58)	0.601
Cerebrovascular disease	296 (21.67)	862 (21.03)	0.619
Valvular heart disease	196 (14.35)	591 (14.42)	0.947
Cancer	235 (17.2)	710 (17.33)	0.918

All data are presented as n (%), unless otherwise indicated.

*SLE* systemic lupus erythematosus, *CCI* Charlson comorbidity index, *AVF* arteriovenous fistula, *HD* hemodialysis, *ACE* angiotensin-converting enzyme, *ARB* angiotensin II receptor blocker, *SD* standard deviation.

^a^Including aspirin, clopidogrel, ticlopidine, and cilostazol.

**Figure 2:**
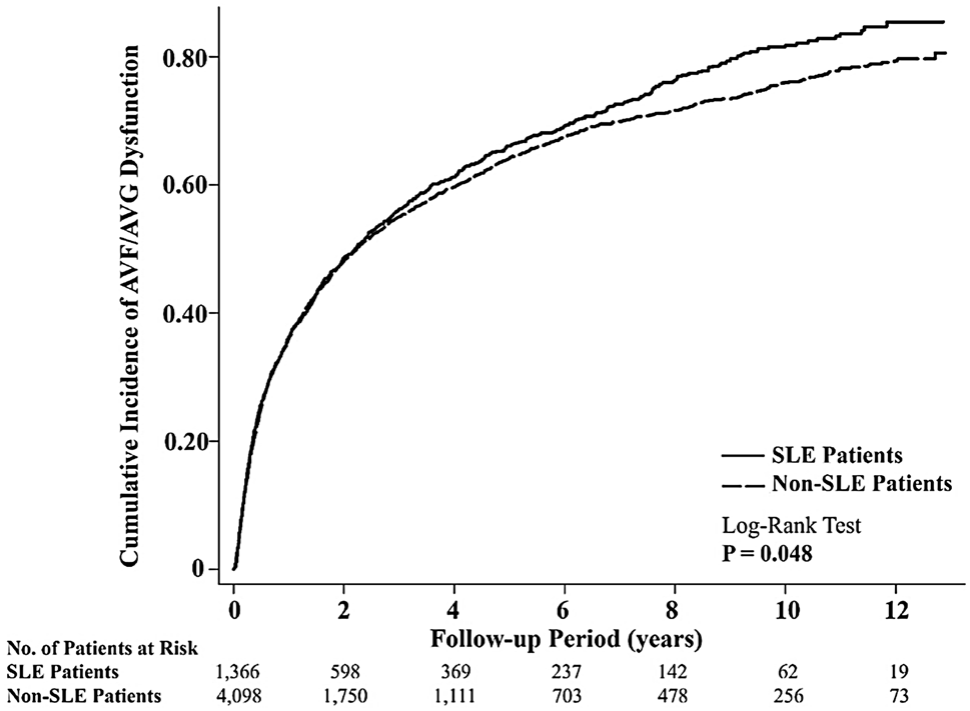
Kaplan–Meier survival estimates showed the cumulative incidence of AVF/AVG dysfunction between SLE and non-SLE patients over 10 years where there was a statistically significant difference between the two groups (P = 0.048). *AVF* arteriovenous fistula, *AVG* arteriovenous graft, *SLE* systemic lupus erythematosus.

The incidence rates (per 100 person-years) and risks of AVF/AVG dysfunction in SLE and non-SLE patients are shown in Table 2. The findings demonstrated that SLE patients had higher incidence rates of AVF/AVG dysfunction than non-SLE patients in all of the specified time periods. There were four specific time periods that reached significant difference: (1) after 1 year (incidence rates = 15.21, 13.01, respectively; subdistribution hazard ratio (SHR) = 1.16; P = 0.007), (2) 1st-to-10th-year period (incidence rates = 15.36 and 13.25, respectively; SHR = 1.16; P = 0.007), (3) 5th-to-10th-year period (incidence rates = 11.91 and 8.1, respectively; SHR = 1.42; P = 0.003), and (4) overall period (incidence rates = 23.53 and 21.66, respectively; SHR = 1.09; P = 0.027). The survival curves in Fig. 2 confirmed these results where there was a statistically significant difference in the cumulative incidence of AVF/AVG dysfunction between SLE and non-SLE patients (P = 0.048). The incidence rates and risks of AVF and AVG dysfunction in SLE and non-SLE patients were also separately analyzed in Table 3. SLE is a significant risk factor for AVF dysfunction (SHR = 1.10; P = 0.022) but not for AVG dysfunction (SHR = 1.0; P = 0.992).

**Table 2:** Incidence rates and risks of AVF/AVG dysfunction in SLE and non-SLE patients.

Time period	SLE patients			Non-SLE patients (reference)			Crude		Competing risk	
	No. of events	Person-years	Incidence rate^a^	No. of events	Person-years	Incidence rate^a^	HR (95% CI)	P	SHR (95% CI)	**P**
Overall period	924	3927	23.53	2589	11,950	21.66	1.07 (0.99, 1.16)	0.068	1.09 (1.00, 1.17)	0.027*
Within 90 days	206	313	65.87	580	937	61.90	1.06 (0.91, 1.25)	0.446	1.06 (0.91, 1.25)	0.442
Within 180 days	347	573	60.52	1009	1720	58.66	1.03 (0.91, 1.17)	0.616	1.04 (0.92, 1.17)	0.572
First year	483	1027	47.05	1434	3071	46.69	1.01 (0.91, 1.12)	0.871	1.01 (0.92, 1.12)	0.782
> 1 year	441	2900	15.21	1155	8879	13.01	1.15 (1.02, 1.29)	0.011	1.16 (1.04, 1.29)	0.007*
1–5 years	332	1973	16.83	910	5824	15.62	1.08 (0.95, 1.22)	0.255	1.09 (0.96, 1.24)	0.174
Within 5 years	815	3000	27.17	2344	8896	26.35	1.03 (0.96, 1.12)	0.4	1.05 (0.97, 1.14)	0.222
5–10 years	100	839.7	11.91	218	2692	8.10	1.47 (1.16, 1.86)	0.001	1.42 (1.12, 1.79)	0.003*
1–10 years	432	2813	15.36	1128	8516	13.25	1.15 (1.03, 1.28)	0.015	1.16 (1.04, 1.29)	0.007*
> 10 years	9	87.7	10.26	27	363	7.45	1.37 (0.64, 2.91)	0.416	1.34 (0.63, 2.85)	0.450

*AVF* arteriovenous fistula, *AVG* arteriovenous graft, *SLE* systemic lupus erythematosus, *HR* hazard ratio, *SHR* subdistribution hazard ratio, *CI* confidence interval.

^a^Per 100 person-years.

**Table 3:** Incidence rates and risks of AVF/AVG dysfunction in SLE and non-SLE patients.

Vascular access type	SLE patients			Non-SLE patients (reference)			Crude		Competing risk	
	No. of Events	Person-years	Incidence rate^a^	No. of Events	Person-years	Incidence rate^a^	HR (95% CI)	P	SHR (95% CI)	**P**
AVF	780	33,724.2	20.94	2173	11,393.9	19.07	1.09 (1.00, 1.18)	0.045	1.10 (1.01–1.19)	0.022
AVG	144	202.7	71.06	416	556.3	74.78	0.94 (0.78, 1.14)	0.540	1.00 (0.83–1.20)	0.992

*AVF* arteriovenous fistula, *AVG* arteriovenous graft, *SLE* systemic lupus erythematosus, *HR* hazard ratio, *SHR* subdistribution hazard ratio, *CI* confidence interval.

^a^Per 100 person-years.

Diabetes mellitus is a crucial underlying cause of ESRD. SLE and non-SLE patients were both further subdivided into groups with or without diabetes mellitus. The incidence rates (per 100 person-years) and risks of AVF/AVG dysfunction between different patient groups are shown in Table 4. Non-SLE patients without diabetes mellitus served as the reference group. The incidence rates of non-SLE and SLE patients without diabetes mellitus were 17.81 and 20.51, respectively, and the difference between groups was found to be statistically significant (SHR = 1.12; P = 0.011). Even if diabetes was removed as a confounding variable, SLE may still directly have an impact on AVF/AVG dysfunction.

**Table 4:** Incidence rates and risks of AVF/AVG dysfunction in SLE and non-SLE patients with and without diabetes mellitus.

Patient group	No. of Events	Person-years	Incidence rate^a^	Crude		Competing risk	
				HR (95% CI)	P	SHR (95% CI)	P
Non-SLE patients without diabetes mellitus	1694	9510	17.81	Reference		Reference	
Non-SLE patients with diabetes mellitus	895	2440	36.68	1.59 (1.47, 1.73)	< 0.001	1.34 (1.23, 1.46)	< 0.001*
SLE patients without diabetes mellitus	614	2994	20.51	1.12 (1.02, 1.23)	0.014	1.12 (1.03, 1.23)	0.011*
SLE patients with diabetes mellitus	310	933	33.22	1.52 (1.35, 1.72)	< 0.001	1.35 (1.20, 1.53)	< 0.001*

*AVF* arteriovenous fistula, *AVG* arteriovenous graft, *SLE* systemic lupus erythematosus, *HR* hazard ratio, *SHR* subdistribution hazard ratio, *CI* confidence interval.

^a^Per 100 person-years.

Approximately 85% of SLE and non-SLE patients enrolled in this study have hypertension. The patients were further subdivided into groups with or without hypertension (Table 5). Within the non-hypertensive group, SLE patients had an SHR of 1.11 (insignificant ratio may be due to an inadequate number of cases), while within the hypertensive group, SLE patients had a subdistribution hazard ratio of 1.08 (which is comparable to the SHR of 1.09 between SLE and non-SLE patients during the overall period in Table 2), which indicates that SLE is still a potent risk factor for AVF/AVG dysfunction than SLE.

**Table 5:** Incidence rates and risks of AVF/AVG dysfunction SLE and non-SLE patients with and without hypertension.

Patient group	No. of Events	Person-years	Incidence rate^a^	Crude		Competing risk	
				HR (95% CI)	P	SHR (95% CI)	P
Non-SLE patients without hypertension	352	2178.8	16.16	Reference		Reference	
Non-SLE patients with hypertension	2237	9771.4	22.89	1.24 (1.10, 1.38)	< 0.001	1.18 (1.06, 1.32)	0.003^a^
SLE patients without hypertension	127	691.3	18.37	1.10 (0.90, 1.35)	0.337	1.11 (0.92, 1.35)	0.283
SLE patients with hypertension	797	3235.6	24.63	1.32 (1.16, 1.50)	< 0.001	1.28 (1.13, 1.44)	< 0.001^a^

*AVF* arteriovenous fistula, *AVG* arteriovenous graft, *SLE* systemic lupus erythematosus, *HR* hazard ratio, *SHR* subdistribution hazard ratio, *CI* confidence interval.

^a^Per 100 person-years.

The incidence rates (per 100 person-years) and risks of MACE, myocardial infarction, and ischemic stroke in SLE and non-SLE patients are shown in Table 6, and the differences between groups were not found to be statistically significant. However, SLE patients have a lower incidence rate of ischemic stroke than non-SLE patients (0.84 vs. 1.09, respectively; SHR = 0.77; P = 0.074) where it may seem that SLE patients have a lower risk of developing ischemic stroke.

**Table 6:** Incidence rates and risks of MACE in SLE and non-SLE patients.

Time period	SLE patients			Non-SLE patients (reference)			Crude		Competing risk	
	No. of Events	Person-years	Incidence rate^a^	No. of Events	Person-years	Incidence rate^a^	HR (95% CI)	P	SHR (95% CI)	**P**
MACE	117	7287	1.61	387	21,196	1.83	0.88 (0.72, 1.08)	0.225	0.89 (0.72, 1.09)	0.26
Myocardial infarction	59	7409	0.8	178	21,674	0.82	0.97 (0.72, 1.30)	0.839	0.98 (0.73, 1.31)	0.89
Ischemic stroke	62	7392	0.84	235	21,534	1.09	0.77 (0.58, 1.02)	0.065	0.77 (0.59, 1.02)	0.074

*MACE* major cardiovascular events, *AVF* arteriovenous fistula, *AVG* arteriovenous graft, *SLE* systemic lupus erythematosus, *HR* hazard ratio, *SHR* subdistribution hazard ratio, *CI* confidence interval.

^a^Per 100 person-years.

## Discussion

The observation period in this study was over a 10-year duration. The results have shown that SLE patients on HD had a significantly higher risk of developing AVF/AVG dysfunction during the long-term period follow up, especially after the first year, 1st-year-to-10th-year, 5th-to-10th-year period, and overall period. To the best of our knowledge, previous research addressing the risk of vascular access dysfunction in SLE patients receiving HD had not analyzed the rate of dysfunction in SLE patients after 1 year^4,6^. The main hypothesis of the study is that there may be a difference in the risk of AVF/AVG dysfunction between SLE and non-SLE patients. The major cause of vascular access failure is venous stenosis as a result of neointimal hyperplasia. Individual variability in the mechanistic response to vascular access maturation and vascular stenosis development after AVF/AVG creation may be influenced by susceptibility factors in SLE. SLE is an autoimmune disease that has micro- and macrovasculature endothelial alterations. The endothelial damage that occurs in SLE patients is related to the persistent inflammatory response and is associated with the presence of autoantibodies, immune complexes, and monocyte activation, which could increase endothelial permeability and complement-dependent cytotoxicity, leading to endothelial activation, apoptosis, and atherogenesis. These responses induce prothrombotic activity and leukocyte recruitment to different tissues, resulting in neointimal hyperplasia^8–11^.

Shafi et al. conducted a study where 66.6% of 36 SLE patients developed VAT at 1 year as compared to 38.9% of 36 non-SLE patients (P < 0.05) and the odds ratio of VAT in SLE patients was 3.1 (95% confidence interval = 1.2, 8.2)^4^. Plantinga et al. carried out a study on 117,836 incident adult and pediatric ESRD patients with a one-year follow-up period and revealed that SLE patients who started treatment with a permanent vascular access on the first dialysis were less likely to experience patency loss than non-SLE patients within the first year (43.8% vs. 55.0%, respectively). This outcome may be due to the nature of the population in this study where SLE-ESRD patients were found less likely to have comorbid conditions (such as diabetes, congestive heart failure, peripheral vascular disease) and to smoke than the other ESRD patients^6^. Cuen-Ojeda et al. conducted a retrospective review of AVFs created between 2008 and 2017 where 134 patients were identified. When compared to patients with chronic diseases such as diabetes, hypertension, and idiopathic ESRD, SLE patients have an increased risk of developing AVF patency loss within the first 6 months of follow-up^12^.

The possible pathogenetic mechanisms associated with a higher risk of vascular access thrombosis (VAT) include the Virchow triad, which consists of stasis, hypercoagulability, and endothelial injury^13^. Stasis is the condition of reduced blood flow within the vascular access. Hypoalbuminemia is a predisposing factor for stasis and is usually attributed to nephrotic syndrome or disease exacerbation in SLE, both of which may lead to vascular access dysfunction^14,15^. Hypercoagulability in SLE may be attributed to lupus-specific antibodies (aPLs), which can lead to VAT through possible mechanisms such as atherogenesis and endothelial activation^4^. Inflammation in SLE may also increase certain procoagulant factors that may increase the risk of developing VAT^16^. Endothelial activation and damage are commonly observed in SLE patients. Different mechanisms have been proposed to explain the prevalence of endothelial dysfunction in SLE^17^. Atehortúa et al. pointed out that different components of the immune system seem to participate in endothelial injury, such as autoantibody production and immune complex formation, which is characterized by an increase in the expression of adhesion molecules, production of pro-inflammatory cytokines and prothrombotic factors, oxidative stress upregulation, and abnormal vascular tone modulation^18^. The structural damage and attenuation of endothelial function in vascular access may lead to their loss of viability and integrity, which may eventually result in a possible long-term vascular access failure.

VAT is a common complication that develops in the majority of HD patients with arteriovenous access, accounting for 65%-85% cases of permanent vascular access loss^19^. aPL antibodies, which include anticardiolipin (aCL) antibodies and lupus anticoagulants (LAC), are the most common acquired blood protein defects causing thrombosis^20,21^. In SLE patients, there were 30%-40% who tested positive for aPL^22^ and the prevalence of positive LAC activity ranged between 11 and 30% and positive aCL activity between 17 and 40%^23–25^.

Grönhangen-Riska et al. reported for the first time the presence of aCL in the HD population^26^. Phillips et al. showed that the aCL presence had no significant relationship with thrombotic events^27^, but Prakash et al. showed that HD patients with elevated IgG-aCL titers have higher odds of recurrent AVG thrombosis^28^. Haviv found that vascular access occluders had higher mean IgG and IgM aCL levels than non-occluders (24.47 and 8.39 IU/mL in occluders [P < 0.0226] vs. 8.45 and 3.59 IU/mL in non-occluders [P < 0.05]). These results indicated that HD patients, especially those with recurrent access occlusion episodes, may be associated with elevated IgG aCL levels, which could be applied to predicting the occlusive status of HD patients^29^. Shafi et al. observed SLE patients on HD during a 1-year period where patients with positive aCL antibodies had a statistically significantly higher rate of VAT (83.3%) as opposed to patients with negative aCL antibodies (33.3%)^4^.

Quereda et al. found that 30% of HD patients exhibited LAC activity compared to 11% of patients on conservative treatment (P < 0.02). Patients with LAC also exhibited a higher incidence of thrombosis than patients without LAC (23% vs. 13%, respectively)^30^. Brunet et al. found that VAT was significantly more frequent in patients with LAC than in patients without LAC (62% vs. 26%, respectively; P = 0.01)^31^. The Lupus in Minorities: Nature vs. Nurture (LUMINA) study found a significant correlation between thrombosis events and shorter disease duration, implying such events occur early in the course of SLE. In addition, the presence of LAC, smoking, older age, disease activity over time, and higher mean daily glucocorticoid dose were identified as risk factors in the development of venous thrombosis^32^. Bataille et al. determined the aPL prevalence and risk factors in 192 HD patients where at least one type of aPL was found in 19.8% of patients, of which 74% had only LAC. There was a significant association between VAT history and aPL presence (hazard ratio = 3.03; 95% confidence interval = 1.69, 4.42; P < 0.001) where aPL presence, especially LAC, is associated with VAT in HD patients^33^.

García-Martín et al. tested both aCL and LAC activity in 51 HD patients where 31% had aCL activity, 22% had LAC activity, and 37% had LAC and/or aCL activity^34^. Wahl et al. conducted a study where patients with SLE and LAC have approximately six times greater risk for venous thrombosis (odds ratio = 5.61; confidence interval = 3.80, 8.27; P < 0.0015) than patients without LAC, whereas patients with SLE and aCL have approximately two times greater risk for venous thrombosis (odds ratio = 2.17; confidence interval = 1.51, 3.11; P < 0.05) than patients without aCL^35^.

SLE patients tend to develop vascular access dysfunction. The presence of comorbidities such as diabetes mellitus and hypertension in SLE patients would further increase the likelihood to experience vascular access problems. This is evidenced in this study where SLE patients with diabetes mellitus presented an SHR of 1.35, while patients with hypertension presented an SHR of 1.28, the highest ratios in their respective tables. Understanding the risk factors that contribute to vascular access problems may lead to regular surveillance and focused care especially in those with SLE superimposed with diabetes mellitus and hypertension, resulting in more effective management of vascular access function.

Numerous studies have shown that SLE patients have increased risks of developing MACE, acute myocardial infarction, and stroke^36–38^. However, this study did not demonstrate SLE patients having a higher risk of developing MACE, AMI, and stroke than non-SLE patients. This may be because the control group was selected using the propensity score matching method with SLE patients concerning the major risk factors for MACE. Furthermore, we evaluated MACE in SLE-HD patients, as opposed to SLE patients only. Therefore, the effect of end-stage renal disease and hemodialysis (HD) may outweigh the effect of SLE on the risk of MACE. The differences in demographic characteristics in the SLE population of this study may account for the different outcomes and further studies may be needed to reevaluate the relationship between SLE, HD, and the aforementioned adverse events. In addition, there was a high percentage of SLE patients who were receiving warfarin compared to non-SLE patients (8% vs. 2%, respectively), and this may explain why SLE patients may seem to have a lower risk of developing ischemic stroke.

There are several limitations in this study that should be addressed. This is a retrospective study and utilized a database where laboratory markers as potential prognostic variables cannot be analyzed. It was also conducted in a single country (Taiwan) where all of the participants were of Chinese ethnicity. The prognosis and outcomes between SLE and non-SLE patients with different ethnicities are unknown, and may limit the generalization of results. The number of patients with AVG listed in the database was too few and thus was combined with the number of patients with AVF for the final analysis. Due to the limited information available in Taiwan’s NHIRD, the type of vascular access dysfunction, vascular anastomosis site, compromised segment (inflow vs. outflow), and vascular access maturation rates were not identified. The percentage of APS cases was not analyzed since there is no specific ICD-9-CM/ICD-10-CM code for APS in the database. The majority of secondary APS cases occur exclusively in association with autoimmune syndromes, especially SLE, and many authors now prefer the term SLE-associated APS. SLE disease activity and damage were also not analyzed based on the limited information in the database. Further research is needed to establish the association between the severity of SLE and vascular access. Despite the aforementioned limitations, this study enrolled the largest number of SLE patients in analyzing vascular access and has the longest observation period of 10 years.

AVF/AVG dysfunction in SLE patients is of crucial clinical relevance since it worsens the quality of life and is a clinical challenge for the healthcare professionals in HD units. Additional randomized large-scale prospective studies are needed in the future to confirm the results in this study and to also address the following important issues: (1) the roles of autoantibodies and other additional factors contributing to the pathogenesis of AVF/AVG dysfunction, (2) the role of antiplatelet or anticoagulation in preventive strategy against VAT, and (3) the interaction between SLE, hemostasis, and immunological system in the pathogenesis of thromboembolism in SLE patients under maintenance HD.

In conclusion, there were significantly higher incidence rates of AVF/AVG dysfunction in SLE patients than non-SLE patients during the long-term follow-up period (especially after 1 year, during the 1st-to-10th year period, and the 5th-to-10th-year period) in this study. Regular surveillance of vascular access function by clinical examinations after 1 year is important, especially during the 5th-to-10th year period, to improve the vascular access patency and dialysis adequacy in SLE patients undergoing maintenance hemodialysis.

## Supplementary Information

Supplementary Table S1.
