# Supplementary material for: Long-term prognosis of vascular access in hemodialysis patients with systemic lupus erythematosus: a retrospective cohort study
Source: Sci Rep. 2021 Jun 15;11:12519. doi: 10.1038/s41598-021-92005-5 (PMC8206131; doi:10.1038/s41598-021-92005-5)
Supplement: Supplementary file 1 — Supplementary Table S1. [file 41598_2021_92005_MOESM1_ESM.docx]

**Long-term Prognosis of Vascular Access in Hemodialysis Patients with Systemic Lupus Erythematosus: A Retrospective Cohort Study**

Fan-Yu Chen^1,2,3^, Chun-Fan Chen^1,2,4^, Ann Charis Tan^3^, Chia-Hao Chan^3^, Fu-An Chen^1,2,4^, Wen-Sheng Liu^1,2,5,6,7,8,9^, Tz-Heng Chen^1,2,10^, Shuo-Ming Ou^1,2,3^, Szu-Yuan Li^1,2,3^, Ming-Tsun Tsai^1,2,3^, Yung-Tai Chen^1,2,9,11*^, Chih-Ching Lin^1,2,3*^

**Institutions:**

^1^School of Medicine, National Yang Ming Chiao Tung University, Hsinchu, Taiwan;

^2^School of Medicine, National Yang-Ming University, Taipei, Taiwan;

^3^Division of Nephrology, Department of Medicine, Taipei Veterans General Hospital, Taipei, Taiwan;

^4^Division of Nephrology, Department of Internal Medicine, National Yang Ming Chiao Tung University Hospital, Yilan, Taiwan;

^5^Division of Nephrology, Department of Medicine, Taipei City Hospital Zhongxing Branch, Taipei, Taiwan;

^6^Institute of Food Safety and Health Risk Assessment, National Yang Ming Chiao Tung University, Hsinchu, Taiwan;

^7^Institute of Food Safety and Health Risk Assessment, National Yang-Ming University, Taipei, Taiwan;

^8^College of Science and Engineering, Fu Jen Catholic University, New Taipei City, Taiwan;

^9^University of Taipei, Taipei, Taiwan;

^10^Division of Nephrology, Department of Medicine, Taipei Veterans General Hospital Fenglin Branch, Hualien, Taiwan;

^11^Division of Nephrology, Department of Internal Medicine, Taipei City Hospital Heping Fuyou Branch, Taipei, Taiwan

***Correspondence to:** Chih-Ching Lin, MD, PhD,

School of Medicine, National Yang-Ming University; Division of Nephrology, Department of Medicine, Taipei Veterans General Hospital,

Address: No. 201, Section 2, Shih-Pai Road, Beitou District, Taipei 11217, Taiwan

Phone: +886-28712121 #2993; E-mail: [lincc2@vghtpe.gov.tw](mailto:lincc2@vghtpe.gov.tw)

*Chih-Ching Lin and Yung-Tai Chen contributed equally to this work as corresponding authors.

**Supplementary Table S1. Propensity Score Model Results of the Probability of SLE Diagnosis**

| **Parameter** | **Estimate** | **Odds Ratio** | **95% CI** | **P** |
| --- | --- | --- | --- | --- |
| Age, per year | -0.0644 | 0.938 | (0.933, 0.942) | <0.0001 |
| Year of Index Date |  |  |  |  |
| 2000 |  | 1 |  |  |
| 2001 | -0.101 | 0.904 | (0.694, 1.177) | 0.4535 |
| 2002 | 0.019 | 1.019 | (0.78, 1.332) | 0.8892 |
| 2003 | 0.0751 | 1.078 | (0.832, 1.397) | 0.5699 |
| 2004 | -0.0216 | 0.979 | (0.749, 1.279) | 0.8747 |
| 2005 | -0.048 | 0.953 | (0.726, 1.251) | 0.7293 |
| 2006 | 0.0686 | 1.071 | (0.812, 1.413) | 0.627 |
| 2007 | 0.0984 | 1.103 | (0.836, 1.457) | 0.4874 |
| 2008 | 0.0451 | 1.046 | (0.792, 1.382) | 0.7507 |
| 2009 | 0.2155 | 1.24 | (0.944, 1.631) | 0.1224 |
| 2010 | 0.1499 | 1.162 | (0.878, 1.537) | 0.2941 |
| 2011 | 0.3079 | 1.361 | (1.022, 1.811) | 0.0347 |
| Month of Index Date |  |  |  |  |
| January |  | 1 |  |  |
| February | 0.1035 | 1.109 | (0.848, 1.451) | 0.4501 |
| March | 0.1604 | 1.174 | (0.905, 1.523) | 0.2273 |
| April | 0.1524 | 1.165 | (0.89, 1.524) | 0.2664 |
| May | 0.1298 | 1.139 | (0.87, 1.49) | 0.3439 |
| June | 0.1738 | 1.19 | (0.908, 1.559) | 0.2079 |
| July | 0.2018 | 1.224 | (0.932, 1.607) | 0.1468 |
| August | 0.1407 | 1.151 | (0.872, 1.519) | 0.3206 |
| September | 0.1851 | 1.203 | (0.916, 1.581) | 0.184 |
| October | 0.1256 | 1.134 | (0.861, 1.493) | 0.3703 |
| November | -0.0258 | 0.974 | (0.734, 1.294) | 0.8583 |
| December | 0.1018 | 1.107 | (0.844, 1.452) | 0.4622 |
| Male | -1.4485 | 0.235 | (0.206, 0.268) | <0.0001 |
| Monthly income |  |  |  |  |
| Dependent |  | 1 |  |  |
| NT 0–19,100 | 0.0159 | 1.016 | (0.865, 1.193) | 0.8458 |
| NT 19,100–42,000 | -0.0483 | 0.953 | (0.835, 1.087) | 0.4727 |
| >NT 42,000 | 0.2229 | 1.25 | (0.934, 1.672) | 0.1331 |
| Urbanization level^a^ |  |  |  |  |
| 1 |  | 1 |  |  |
| 2 | -0.1826 | 0.833 | (0.742, 0.935) | 0.002 |
| 3 | -0.3226 | 0.724 | (0.54, 0.972) | 0.0315 |
| 4 | -0.9426 | 0.39 | (0.171, 0.889) | 0.025 |
| CCI score | 0.3527 | 1.423 | (1.374, 1.473) | <0.0001 |
| AVF | -0.126 | 0.882 | (0.739, 1.052) | 0.1615 |
| Concomitant medications |  |  |  |  |
| Antiplatelet agents^‡^ | 0.0127 | 1.013 | (0.893, 1.148) | 0.8433 |
| ACE inhibitor or ARB | 0.1664 | 1.181 | (1.046, 1.334) | 0.0074 |
| Beta blocker | 0.0554 | 1.057 | (0.934, 1.196) | 0.3786 |
| Calcium channel blocker | 0.155 | 1.168 | (1.021, 1.335) | 0.0232 |
| Statin | 0.0477 | 1.049 | (0.876, 1.256) | 0.6036 |
| Comorbidities |  |  |  |  |
| Diabetes mellitus | -1.6009 | 0.202 | (0.168, 0.242) | <0.0001 |
| Hypertension | -0.0206 | 0.98 | (0.816, 1.175) | 0.8246 |
| Myocardial infarction | -0.2989 | 0.742 | (0.567, 0.97) | 0.0294 |
| Heart failure | -0.2878 | 0.75 | (0.655, 0.858) | <0.0001 |
| Peripheral vascular disease | -0.3822 | 0.682 | (0.544, 0.856) | 0.001 |
| Dementia | -0.1614 | 0.851 | (0.59, 1.226) | 0.3867 |
| Chronic pulmonary disease | -0.0452 | 0.956 | (0.843, 1.084) | 0.4801 |
| Dyslipidemia | 0.0282 | 1.029 | (0.901, 1.174) | 0.6761 |
| Cerebrovascular disease | -0.4397 | 0.644 | (0.551, 0.753) | <0.0001 |
| Valvular heart disease | 0.1791 | 1.196 | (1.014, 1.41) | 0.0332 |
| Cancer | -0.9215 | 0.398 | (0.321, 0.493) | <0.0001 |

^a^Urbanization levels in Taiwan are divided into four strata according to the Taiwan National Health Research Institute publications. Level 1 designates the most urbanized areas, and level 4 designates the least urbanized areas.

^b^Charlson Comorbidity Index (CCI) score is used to determine overall systemic health. With each increased level of CCI score, there are stepwise increases in the cumulative mortality.[_._](file:///E:\Data%20D%20VGH\Scientific%20Reports\REVISION%232%20YTC\FINAL\Prognosis%20of%20vascular%20access%20in%20HD%20patients%20with%20ADPKD.docx#_ENREF_9)

^‡^Including aspirin, clopidogrel, ticlopidine, and cilostazol

Abbreviations: SLE, systemic lupus erythematosus; NT$, New Taiwan dollars; CCI, Charlson Comorbidity Index; AVF, arteriovenous fistula; ACE, angiotensin-converting enzyme; ARB, angiotensin II receptor blocker; CI, confidence interval
